# Promotion and prelacteal feeding of breastmilk substitutes among mothers in Kathmandu Valley, Nepal

**DOI:** 10.1111/mcn.12205

**Published:** 2016-04-15

**Authors:** Alissa M. Pries, Sandra L. Huffman, Indu Adhikary, Senendra Raj Upreti, Shrid Dhungel, Mary Champeny, Elizabeth Zehner

**Affiliations:** ^1^ Helen Keller International Asia Pacific Regional Office Phnom Penh Cambodia; ^2^ Consultant to Helen Keller International; ^3^ Helen Keller International Kathmandu Nepal; ^4^ Ministry of Health and Population Kathmandu Nepal; ^5^ Helen Keller International Washington, DC USA

**Keywords:** Nepal, prelacteal feeding, breastfeeding, breastmilk substitutes

## Abstract

In 1992, Nepal passed the *Mother's Milk Substitutes (Control of Sale and Distribution) Act* in order to regulate the sale, distribution and promotion of substitutes for breastmilk within Nepal, in an effort to protect and promote breastfeeding. Helen Keller International, in collaboration with Nepal's Ministry of Health and Population's Child Health Division, implemented a study to assess mothers' exposure to promotions for and utilization of breastmilk substitutes in Kathmandu Valley, Nepal. A health facility‐based, cross‐sectional survey was conducted among 304 mothers being discharged after delivery. Prelacteal feeding of breastmilk substitutes is prevalent (55.9% of mothers, *n* = 170). Reported recommendations during antenatal checks and after delivery from health professionals to use breastmilk substitutes were prevalent, occurring among 47.4% (*n* = 144) of mothers; rates of these recommendations were significantly higher for mothers that delivered in private health facilities, as compared with public (67.7% vs. 38.0%, *P* < 0.001). Mothers that received a recommendation to use a breastmilk substitute from a health worker were 16.7 times more likely to provide a prelacteal feed of a breastmilk substitute, as compared with mothers that did not receive a recommendation (*P* < 0.001). Few mothers reported observation of commercial advertisements for breastmilk substitutes inside a health facility (reported by 3.6% of mothers). No mothers reported receiving a sample of a breastmilk substitute, bottle or teat from a health professional. More information is needed to determine why there is such a high rate of health worker recommendations for breastmilk substitute use in the first few days after delivery.

**Key messages**

- While utilization of breastmilk substitutes is low among mothers of young children in Nepal, prelacteal feeding of breastmilk substitutes is highly prevalent in Kathmandu Valley.
- Reported recommendations from health professionals to use breastmilk substitutes are common (over 40%) and are associated with prelacteal feeding among Nepal mothers included in this study.
- Provision of lactation management training to health workers and monitoring their practices regularly could strengthen breastfeeding counselling, aid in reducing high rates of prelacteal feeding and contribute to improved infant feeding practices.

## Introduction

The initiation of breastfeeding within the first hour after delivery is associated with the reduced risk of neonatal morbidity and mortality (Debes *et al*. 2013) and improved maternal health outcomes (Sobhy & Mohame 2004). Exclusive breastfeeding for the first 6 months of life with continued breastfeeding up to 2 years of age or beyond is the optimal course of feeding for infants and young children (World Health Organization (WHO) and United Nations Children's Fund (UNICEF) 2003); exclusive and continued breastfeeding hold vital importance for reducing mortality rates for children less than 5 years of age (Black *et al*. 2013). Immediate skin‐to‐skin contact between mothers and newborns after delivery and the provision of only breastmilk to newborns within the first few days after delivery have been shown to support longer‐term breastfeeding outcomes, specifically the duration of mothers' exclusive breastfeeding (Moore *et al*. 2012). Breastfeeding is prevalent in Nepal, where 70% of infants under 6 months of age are exclusively breastfed (Ministry of Health and Population (MOHP) 2012). Continued breastfeeding up to 24 months of age is also high; 93% of infants 12–15 months of age are breastfed in Nepal, with the rate of breastfeeding remaining high for children 21–23 months of age. However, prelacteal feeding is common; approximately 28% of Nepalese children have been fed something other than breastmilk in the first 3 days after birth (MOHP 2012).

The Nepali government has affirmed its commitment to support optimal breastfeeding practices. In 1992, Nepal passed its own national legislation for commercial infant and young child food products, the *Mother's Milk Substitutes (Control of Sale and Distribution) Act*. The Act was passed to regulate the sale, distribution and promotion of substitutes for breastmilk within Nepal, including breastmilk substitutes and ‘any other such food or beverage marketed or otherwise distributed as is suitable for feeding to the infant’ (Nepal Government 1992). However, because the Act defines an ‘infant’ as a child under 12 months of age, this policy only applies to breastmilk substitutes and commercial complementary food products marketed for children less than 12 months of age.

Specific provisions with regard to the health care system are outlined in Clause 8 of the Act. Health care workers must promote proper breastfeeding practices and must be familiar with the provisions of the *Mother's Milk Substitutes Act* and the information it contains. Health care workers may not accept gifts or incentives from manufacturers or distributors of breastmilk substitutes, distribute product samples or promote the use of breastmilk substitutes. Additionally, Clause 9 of the Act states that manufacturers and distributors should not advertise or promote any product outside or inside a health facility; advertisement in the Act includes: television, radio, film, telephone, symbols, billboards or the exhibition of images, materials or product information (Nepal Government 1992).

Understanding what messages mothers in Kathmandu Valley receive from the health system and outside from the commercial sector about infant feeding is needed in order to reinforce positive messages and discourage inappropriate promotion of commercially produced foods for infants and young children, including breastmilk substitutes. It is also important to ensure that breastmilk substitutes are not being promoted, which would be in violation of the International Code of Marketing of Breast‐milk Substitutes (WHO 1981) and Nepali law (if the product is for children under 1 year of age). Research regarding Code compliance has not yet been conducted within Nepal, and a recent Baby‐Friendly Hospital Initiative (BFHI) assessment indicated that none of hospitals that were certified within Kathmandu Valley remain fully compliant (Shrestha *et al*. 2012).

Helen Keller International, in partnership with Nepal's Ministry of Health and Population's Child Health Division, conducted a study to assess mothers' exposure to commercial promotions for infant and young child food products in Kathmandu Valley and their utilization of these products. Specifically, the objectives of the research were to estimate the prevalence of promotional practices occurring within the health system for breastmilk substitutes, including infant formula, follow‐on formula, and growing‐up/toddler milks, document breastfeeding support and counselling provided in health facilities, and document consumption of breastmilk substitutes among infants and young children in Kathmandu Valley.

## Materials and methods

### Research design and study population

This study was a cross‐sectional survey using a multi‐stage sampling procedure to obtain a representative sample of Kathmandu Valley mothers discharged after delivery in health facilities. Because variables of interest included breastfeeding practices, the study was limited to only mothers and did not include other caregivers of children. Data were collected through structured interviews among mothers who had just been discharged from a maternity ward after delivery. Recently delivered mothers were interviewed regarding experiences and practices during their pregnancy or since the recent delivery of their newborn. Data were gathered from a period of December 2013–February 2014.

Because prior studies have indicated that urban infants are more likely than those in rural areas to consume commercial infant and young child feeding (IYCF) products (Huffman *et al*. 2014), the study population included in this survey was limited to mothers currently living in and utilizing health facilities within Kathmandu Valley, defined as the geographical area within the limits of Kathmandu, Lalitpur and Bhaktapur districts. Mothers living outside of Kathmandu Valley, but utilizing delivery or child health services in the Valley, were excluded from participation in the survey. Mothers with any of the following characteristics were excluded because these conditions held the potential to delay breastfeeding initiation: mothers of newborns with congenital diseases or who were in the neonatal intensive care unit; mothers who experienced severe delivery complications during the birth of their newborn; mothers whose newborn is a twin or from a multiple birth; newborns too ill for their mothers to be interviewed.

### Sample size

The IGBM and UNICEF (2005) methodology uses 800 women with infants<6 months because ‘The sampling of 800 women gives a 95% power to observe at least one reported violation if the true prevalence is 2%. If the prevalence is 10%, the sample size generates an estimate of population prevalence with a standard error of 1%.’ Because of the high cost of collecting such a large sample size and the desire to develop a methodology that can be replicated in subsequent years on a regular basis, a higher standard error (2.55%) was used in this study. The sample size for this study was calculated to detect a 10% prevalence rate of exposure to promotions within the health system, with a measurement error of ±5%. Using a standard of error of 0.0255 and assuming a design effect of 2 to account for the cluster design, a sample size of 280 mothers at discharge was determined. Because of the cluster sampling design utilized (described later), the final sample size was slightly higher than 280. A total of 452 mothers being discharged after delivery were approached for interview. Twenty‐six (5.8%) mothers refused participation and 122 (27.0%) were excluded based on at least one of the criteria detailed previously; 101 (22.3%) of the mothers lived outside of Kathmandu Valley, 20 (4.4%) infants had been in the neonatal intensive care unit after delivery, 8 (1.8%) mothers reported severe complications during delivery, and 2 (0.4%) children were from a multiple birth. The final sample of successfully completed interviews among mothers discharged after delivery was 304 mothers.

### Sampling procedure and data collection

In order to reflect the significant share of Nepal's urban mothers who utilize private facilities, a proportion of the sample for each study population was taken from private facilities; approximately 28% of urban Nepali mothers with facility‐based deliveries deliver at private facilities (MOHP 2012). Therefore, 30% of the total sample of mothers were interviewed at private facilities. Rates of refusal and exclusion were higher at public facilities (7.1% and 31.0%, respectively) as compared with private facilities (1.7% and 15.5%, respectively).

Lists of all health facilities, including both public and private, offering maternity were obtained from the Health Management Information System database from the District Public Health Offices for the three districts in Kathmandu Valley. This included national hospitals, referral hospitals and health centres; health posts were excluded. Additionally, this same data source contained utilization rates for these facilities, which included the total number of deliveries. Health facilities were then sampled by allocating clusters using probability proportional to size, with the calculated monthly delivery rates serving as each facility's ‘population’. Because of the logistics requirements and the desire to complete data collection within 8–10 weeks, facilities with less than 50 deliveries per month were excluded from the sampling frame. This excluded 40 out of 48 facilities for delivery; however, the eight included in the sampling frame represented 91.0% of all facility‐based births in Kathmandu Valley that occurred between July 2012–July 2013.

**Figure 1:**
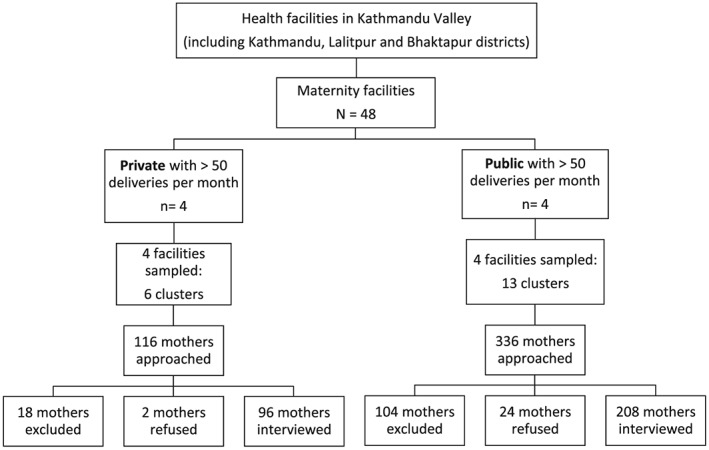
Sampling profile for mothers and facilities

Clusters of 16 mothers each were assigned across facilities in the sampling frame. Thirteen clusters were sampled in the sampling frame of public facilities and six clusters were sampled for private facilities, allowing for 30% of the total sample to be sourced from private facilities. Because sampling of facilities was proportional to size, larger facilities had a greater chance of being sampled for multiple clusters, while smaller facilities had a greater chance of being sampled for only one cluster. Figure 1 details the sampling of facilities and mothers for each study population across public and private health facilities.

Sampled facilities were alerted of data collection approximately 1 week prior to survey. Survey supervisors worked closely with nurses in charge to identify those mothers ready for discharge, and enumerators interviewed mothers after they had completed the discharge procedure and paperwork. Interviews continued until all mothers scheduled for discharge that day had been interviewed or until the sample size for the facility in question had been reached.

Approval for this study was obtained from the Nepal Health Research Council prior to data collection. 1Approval was obtained on December 2, 2013 (Registration No. 156/2013). Informed consent was obtained from all participants prior to the conducting of any interview.

### Questionnaire design

The questionnaire collected data on mothers' and newborns' characteristics, including mother's age, marital status, caste, educational attainment, household assets and drinking water source, details regarding antenatal care and delivery of the newborn, and the newborn's date of birth, gender and birth order. Data on breastfeeding initiation, prelacteal feeding and current breastfeeding practices for the newborn were also collected. Data to assess these infant and young child feeding practices were gathered in accordance with the World Health Organization guidelines on IYCF practices (WHO 2008). Mothers were also asked to report on commercial promotional practices experienced inside and outside the health system during pregnancy and after delivery of their newborn.

Data were collected using a mobile technology system in order to allow for immediate data entry, reduction in data errors, and prompt analyses. The questionnaires were designed in Microsoft Word and then entered in Formhub, an open‐source online platform that allowed data to be collected *via* tablets, using the Android application Open Data Kit (ODK) Collect, and data were then submitted online to a web‐based database (Formhub.org 2013). The questionnaires were translated from English into Nepali, back translated into English to ensure accuracy, and uploaded into Formhub in Nepali. Interviews were conducted in Nepali using the Samsung Galaxy tab 2.07 model tablet. Submitted questionnaires were reviewed weekly to ensure data quality.

### Statistical analyses

Data were cleaned and analyzed using spss version 21 (IBM, Armonk, NY, USA) and stata version 11 (StataCorp LP, College Station, Texas, USA). Proportions and mean ± standard deviation were used to describe the sample. Differences in age categories and associations were assessed through bivariate comparison, using two‐sided Pearson's chi‐square test for proportions and through multivariate analysis, using logistic regression.

This study defined breastmilk substitutes to include infant/starter formula (to be used from birth up to 6 months of age), follow‐up formula (to be used from 6 months to 12 months), infant/follow‐up formula for special dietary or medical purposes, growing‐up milk (to be used from 12 months to 36 months) and other milk or milk‐like products (in liquid or powdered form) marketed or otherwise represented as suitable for feeding children younger than 6 years of age. Mothers were also asked to report brands of breastmilk substitutes mentioned in order to verify that these products fit this definition.

Commercial promotions were defined as any type of marketing technique intended to increase sales, including media or print advertising, provision of free samples or any other activity to encourage or induce the purchase of a product (International Baby Food Action Network (IBFAN) 2007). Exposure to such commercial promotion within the health system was measured by asking mothers if they had heard, seen or read any promotions during pregnancy or since the delivery of their newborns, and if so, where they observed the promotion. Mothers were also asked if they had received any free samples of products, and if so, where they received the sample. Additionally, mothers were asked if they had observed any display of logos or brands on equipment or materials within a health facility. Interpersonal promotions were defined as recommendations/advice from a health professional to use a breastmilk substitute; measurement of exposure to interpersonal promotion was obtained by asking mothers if they had received such a recommendation during pregnancy or since the delivery of their newborns. The utilization of breastmilk substitutes was defined and measured as a mother's reported use of a product as a prelacteal feed with her newborn, meaning within the first 3 days after delivery.

Multivariate logistic regression was conducted to assess the impact of variables shown to influence prelacteal feeding of breastmilk substitutes among mothers during bivariate analysis. Covariates included in the model were mother's age in years, attainment of secondary or higher level of education, vaginal vs. caesarean delivery and receipt of a recommendation from a health worker to use breastmilk substitutes. The model was also adjusted for cluster sampling. Goodness‐of‐fit for this model was determined through the Hosmer–Lemeshow test, with a significance of *P* = 0.494.

## Results

### Demographics and socioeconomic characteristics

Demographic and socioeconomic characteristics for mothers of newborns discharged after delivery are shown in Table 1. The majority of all mothers were currently married at the time of interview, and almost two‐thirds (62.2%, *n* = 189) of mothers reported their newborn to be their only child. Among those that were currently married, 14.5% (*n* = 44) of mothers reported that their husband currently worked outside of Kathmandu. Ninety per cent of mothers had attended any level of formal education and 19.1% (*n* = 58) of mothers reported attending university or higher graduate studies. Only 15.1% (*n* = 46) of mothers reported currently working outside the home.

**Table 1:** Demographic and socio‐economic characteristics

	Mothers discharged after delivery (*n* = 304)
Mother	
Age (years) (mean ± SD)†	25.0 ± 4.6
Parity (number) (mean ± SD)	1.5 ± 0.6
Marital status (%)	
Married	99.7 (303)
Divorced, widowed or separated	—
Single	0.3 (1)
Level of education (%)	
None	7.6 (23)
Non‐formal education	2.3 (7)
Primary	19.7 (60)
Secondary	28.0 (85)
Upper secondary	23.4 (71)
Tertiary education	19.1 (58)
Caste (%)	
Dalit	3.6 (11)
Disadvantaged janajati	31.3 (95)
Disadvantaged non‐dalit terai caste	1.0 (3)
Religious minority	0.0 (0)
Advantaged janajati	30.3 (92)
Upper caste	33.9 (103)
Works outside the home (%)	15.1 (46)
Received antenatal care (%)	99.3 (302)
Assisted delivery (%)	99.0 (301)
Child	
Age (mean ± SD)	2.2 ± 2.5 (days)
Sex (female) (%)	43.8 (133)
C‐section delivery (%)	29.3 (89)
Household	
Safe source of drinking water (%)	95.7 (291)
Household members per sleeping room (mean ± SD)	2.7 ± 1.0
Assets, ownership (%)	
Bicycle	23.0 (70)
Car	6.3 (19)
Motorbike	45.4 (138)
Refrigerator	39.1 (119)
Television	86.2 (262)

† SD, standard deviation.

Just over half (56.2%, *n* = 171) of the newborns included in this survey were male. The mean age of newborns referred to in interviews with mothers discharged after delivery was 2.2 days. Almost all mothers had received some antenatal care (ANC) during pregnancy with their newborn; 93.7% of women living in urban Nepal had received ANC according to the most recent Nepal Demographic and Health Survey (NDHS) (MOHP 2012). Approximately one‐third of the newborns referenced in this survey were delivered by caesarean section and almost all mothers delivered their newborn with the assistance of a health professional, including a doctor, nurse or auxiliary nurse midwife. According to the 2011 NDHS, the rate of C‐section deliveries across urban Nepal is 22.3% (MOHP 2012).

A comparison of these characteristics to the 2011 NDHS indicates that the mothers included in this study are of a slightly higher socioeconomic status than general women in urban Nepal. Seventy per cent of mothers in this study had achieved an educational level of secondary school or higher, as compared with 63.7% of urban Nepal women, and only 7.6% of mother included in the survey had never attended school, as compared with 22.0% of women living in urban Nepal (MOHP 2012). Household assets were also greater among mothers included in this survey. Nearly half of mothers (45.4%) reported that their household owned a motorbike, 39.1% a refrigerator and 86.2% a television, as compared with 27.8%, 29.3% and 76.2% of urban Nepal women, respectively (MOHP 2012).

Several differences in demographic and socioeconomic characteristics were found when comparing mothers attending public vs. private health facilities in Kathmandu Valley. Ownership of refrigerators, motorbikes and cars was higher among mothers attending private health facilities, as compared with those attending public facilities (data not shown). Mothers attending private facilities reported higher rates of C‐section deliveries as compared with mothers attending public facilities; 40.6% of mothers from private maternity wards delivered *via* C‐section, as compared with 24.0% of mothers from public maternity wards (*P* = 0.004).

### Breastfeeding counselling and educational messaging

Information was gathered from participants in order to assess mothers' experience of advice and counselling within health facilities that would support and encourage optimal breastfeeding and complementary feeding practices. Mothers discharged after delivery in a maternity ward were asked about their exposure to breastfeeding counselling and messages throughout their pregnancy and during delivery (results are shown in Table 2).

**Table 2:** Breastfeeding messaging during antenatal care

	Mothers discharged after delivery (*n* = 304)
Receiving information on breastfeeding from a health worker during ANC (%)†	11.6
Breastfeeding messages received during ANC (%)‡	
Exclusive breastfeeding	62.9 (22)
Early initiation	51.4 (18)
Feeding colostrum	25.7 (9)
Frequent breastfeeding (8–12 times a day)	22.9 (8)
Continued breastfeeding until 2 years and beyond	14.3 (5)
Demand breastfeeding	5.7 (2)
Risks of feeding infant formula	2.9 (1)
Risks of feeding other foods/liquids before 6 months	2.9 (1)
Increased breastfeeding during/after illness	0.0 (0)

ANC, antenatal care.

† Among discharged mothers that received antenatal care during pregnancy with newborn (n = 298);

‡ Among discharged mothers that received breastfeeding information during ANC pregnancy with newborn (n = 35).

Among mothers discharged after delivery who received ANC during this pregnancy, 11.6% (*n* = 35) reported receiving breastfeeding information during an ANC visit. The most commonly reported breastfeeding messages received among these mothers were related to promotion of exclusive breastfeeding and early initiation of breastfeeding. Messages regarding the risks of feeding infant formula, the risks of feeding other foods/liquids before 6 months, and increasing breastfeeding during and after illness were the least commonly reported information received by mothers. Approximately two‐thirds (64.5%, *n* = 196) of mothers reported receiving assistance in positioning and/or attachment for breastfeeding from a health worker after delivery of their newborn. Rates of assistance with positioning/attachment were significantly higher in private health facilities as compared with public (83.3% vs. 55.1%, *P* < 0.001).

### Promotion of breastmilk substitutes within the health system

In addition to gathering information regarding breastfeeding advice and counselling received by mothers in the health system, mothers were also asked about recommendations received from health professionals for use of breastmilk substitutes and exposure to other commercial promotions for breastmilk substitutes within the health system (results are shown in Table 3).

**Table 3:** Exposure to breastmilk substitute promotions within the health system

	Mothers discharged after delivery (*n* = 304)
Received recommendation from a health professional	47.4 (144)
Observed branding/logos on health facility equipment	4.9 (15)
Observed commercial advertisement within health facility	3.6 (11)
Received sample from a health professional	0.0 (0)
Received a branded gift from a health professional	0.0 (0)

Recommendations for breastmilk substitute use by health professionals were commonly reported by mothers that had been discharged after delivery. Nearly half (47.4%, *n* = 144) received a recommendation from a health professional, either during pregnancy or just after delivery, to feed their newborn breastmilk substitutes. The majority of these mothers reported recommendations by health professionals to use a breastmilk substitute in the first 3 days after delivery of their youngest child; health professional recommendations to use infant formula for prelacteal feeding were reported by 40.8% (*n* = 124) of mothers. Nurses made the majority of recommendations (81.9%, *n* = 118) for breastmilk substitutes to mothers, with doctors accounting for the remaining recommendations. Rates of breastmilk substitute recommendations from health professionals were significantly higher for mothers that delivered in private health facilities, as compared with public (67.7% vs. 38.0%, *P* < 0.001). Among those that received a health professional recommendation to feed their newborn breastmilk substitute, around one‐third (31.5%, *n* = 34) of mothers were recommended to feed a specific brand of breastmilk substitute. The majority of these brand recommendations were for Nestle Lactogen, accounting for 91.2% (*n* = 31) of brand recommendations.

Observation of commercial advertisements for breastmilk substitutes within the health system was not common among mothers interviewed; only 3.6% (*n* = 11) of mothers discharged after delivery reported seeing, hearing or reading such an advertisement within a health facility during pregnancy or since delivery of their newborn. Slightly more mothers attending public health facilities (4.8%, *n* = 10) reported observation of a breastmilk substitute advertisement within a health facility, as compared with those attending private facilities (1.0%, *n* = 1); however, this difference was not significant (*P* = 0.165). Nine of the 10 health facility advertisements reported were for Nestle Lactogen brand. Mothers reporting observations of breastmilk substitute or commercially produced complementary food branding or logos on health facility equipment or materials were also rare, reported by 15 (4.9%) of the 304 mothers. Almost all cases of observed branding were reported to have occurred on posters displayed in a health facility.

No mothers reported receiving such a sample of a breastmilk substitute, bottle, pacifier or teat from a health professional; however, 17 (5.6%) and 26 (8.6%) mothers reported receiving a gift of diapers or baby clothes, respectively, from a health professional. However, none of the mothers reported any commercial product branding present on these gifts.

### Early breastfeeding practices

Breastfeeding initiation and practices by mothers soon after delivery were documented among mothers (findings are shown in Table 4). Over one‐third of mothers (40.8%, *n* = 124) initiated breastfeeding early, defined as within one hour after delivery (WHO 2008). Early initiation of breastfeeding was significantly higher among mothers who delivered at public facilities (52.4%, *n* = 109), as compared with private (15.6%, *n* = 15; *P*‐value < 0.001). Immediate initiation of skin‐to‐skin contact with the newborn was low, occurring among less than 9% (*n* = 26) of mothers who had just delivered. Prelacteal feeding, referring to the provision of liquids other than breastmilk in the first 3 days after delivery, was prevalent, with over half of mothers discharged after delivery reporting prelacteal feeding for their newborn. Rates of prelacteal feeding among mothers discharged after delivery in public facilities (48.6%, *n* = 101) were found to be significantly lower, as compared with private facilities (74.0%, *n* = 71; *P*‐value < 0.001). Additionally, more mothers who delivered by C‐section reported provided a prelacteal feed, as compared with mothers who delivered vaginally (89.9% vs. 42.8%, *P* < 0.001).

**Table 4:** Early breastfeeding practices among mothers, comparing public vs. private facilities

	Total (*n* = 304)	Public (*n* = 208)	Private (*n* = 96)	*P*‐value
Ever put to the breast, %	99.3 (302)	99.5 (207)	99.0 (95)	0.268
Early initiation of breastfeeding, %	40.8 (124)	52.4 (109)	15.6 (15)	<0.001
Immediately held newborn after birth, %	8.6 (26)	10.1 (21)	5.2 (5)	0.189
Prelacteal feeding, %	56.6 (172)	48.6 (101)	74.0 (71)	<0.001

Among mothers who gave their newborn something to drink other than breastmilk in the first 3 days after delivery, breastmilk substitute was the most commonly used liquid for prelacteal feeding. Ninety‐nine per cent (*n* = 170) of mothers discharged after delivery who practiced prelacteal feeding fed their newborn a breastmilk substitute. Plain water (not bottled) was the second most common liquid used for prelacteal feeding; however, this was only reported among 4.1% (*n* = 7) of discharged mothers. Approximately three‐quarters (73.5%, *n* = 125) of mothers who gave a breastmilk substitute as a prelacteal feed reported using Nestle Lactogen brand, while approximately one‐quarter (25.9%, *n* = 44) fed their newborn Wockhardt Farex brand. Almost all (90.6%, *n* = 154) mothers that fed a breastmilk substitute in the first 3 days after delivery obtained the breastmilk substitute by purchase, vs. receiving it for free or from another mother in the delivery ward. As detailed previously, health professionals, specifically doctors and nurses, were reported as recommending breastmilk substitutes by the majority of mothers who practiced prelacteal feeding.

Although prelacteal feeding of breastmilk substitutes was high among mothers interviewed, many mothers reported that this was not actually what they wanted for their newborn. Nearly two‐thirds (59.4%, *n* = 101) of mothers who provided breastmilk substitutes as a prelacteal feed reported having not wanted breastmilk substitutes fed to their newborn. The most common reasons mothers reported were related to their own breastmilk supply. Over half of mothers that gave a prelacteal feed (54.1%, *n* = 92) reported using a breastmilk substitute because their own breastmilk supply was low and around one‐third (27.1%, *n* = 46) reported prelacteal feeding a breastmilk substitute because their breastmilk had not yet come in. There was no statistical association between particular reasons mothers reported using a breastmilk substitute as a prelacteal feed and their receipt of a health worker's recommendation to use breastmilk substitute (data not shown). Mothers who reported that they thought their own breastmilk supply was low were asked why they believe this was so; the majority of these mothers reported their own breastmilk supply as low because of their poor health or nutrition. Several (0.7%, *n* = 2) also reported that lactation is generally poor on the first day after delivery, and some (2.0%, *n* = 6) reported their milk supply to be low because it was their first child.

### Associations between promotion and consumption of commercially produced foods

In bivariate analyses, 88.2% (*n* = 127) of discharged mothers who received a recommendation to use a breastmilk substitute from a health professional reported feeding a prelacteal, as compared with 26.9% (*n* = 43) of discharged mothers who did not receive a health professional's recommendation (*P* < 0.001).

Results of multivariate analysis are shown in Table 5. Delivery by caesarean section and receiving a recommendation from a health professional to use a breastmilk substitute were found to be the most influential factors in mothers' provision of breastmilk substitutes as a prelacteal feed. Mothers that delivered their newborns *via* caesarean section were 8.8 times more likely to prelacteal feed as compared with mothers that had a vaginal delivery (*P* < 0.001). Mothers that reported receiving a health professional's recommendation to use a breastmilk substitute were 16.7 times more likely to provide this product as a prelacteal feed to their newborns as compared with mothers that did not receive a recommendation (*P* < 0.001).

**Table 5:** Multivariate logistic regression of predictive variables for prelacteal feeding among mothers (*n* = 304)

	*P*‐value	Odds ratio	95% confidence interval
Mother age (years)	0.182	1.02	(0.99–1.05)
Maternal educational attainment	<0.001	1.91	(1.45–2.53)
Caesarean delivery	<0.001	8.80	(3.21–24.09)
HW recommendation for breastmilk substitute	<0.001	16.71	(4.77–58.54)

## Discussion

Study findings suggest that almost half of women residing in Kathmandu Valley who give birth in one of eight large health facilities in Kathmandu Valley receive a recommendation from a health professional to use a breastmilk substitute during their pregnancy or since delivery. Those receiving such a recommendation and/or who give birth by C‐section are 8.8 to 16.7 times more likely to feed their newborn a breastmilk substitute as a prelacteal. Mothers' exposure to commercial advertisements promoting breastmilk substitutes within the health system was low.

Clause 8.3 of Nepal's *Mother's Milk Substitutes Act* states that ‘*health workers shall not allow any act inhibiting the commencement and expansion of breastfeeding, whether directly or indirectly*’ and Clause 9.5 prohibits promotion by manufacturers within any health facilities (Nepal Government 1992). Findings from this study indicate that promotion of breastmilk substitutes within the Kathmandu Valley health system occurs mainly through recommendations by health professionals, nurses and doctors, but these products are rarely commercially promoted in facilities through advertisements, distribution of samples or branding of equipment or gifts. Almost half of mothers at discharge after delivery (47.4%, *n* = 144) reported receiving a recommendation from a health professional during pregnancy or since delivery to feed their newborn a breastmilk substitute.

Prelacteal feeding of breastmilksubstitutes was highly prevalent; 55.9% (*n* = 170) of mothers at discharge after delivery reported feeding their youngest child a breastmilk substitute in the first 3 days after delivery. Despite increasing rates of early initiation of breastfeeding, the practise of prelacteal feeding is prevalent among mothers in Nepal and has been increasing over the last decade (MOHP 2007, 2012). Other research has confirmed this finding; Khanal *et al.* found that 39% of mothers in Kapilvastu district in Western Nepal had given a prelacteal feed to their newborn (Khanal *et al*. 2013b), while the most recent NDHS found that 28% of urban mothers in Nepal gave a prelacteal feed to their youngest child (MOHP 2012). Given that prelacteal feeding hinders the practice of exclusive breastfeeding, can reduce the duration of breastfeeding and carries an increased risk of infection, this finding carries great weight for the protection and promotion of optimal breastfeeding in Kathmandu Valley (Sundaram *et al*. 2013). Furthermore, the implications of these high rates of prelacteal feeding on measurement of exclusive breastfeeding rates in Nepal should be considered.

In contrast to this finding, another study conducted among older infants in Kathmandu Valley (Pries *et al.* 2016) found that consumption of breastmilk substitutes was low among children less than 24 months of age; only 6.2% of children 0–5 months and 7.5% of children 6–23 months had consumed a breastmilk substitute in the day prior to interview. Additionally, continued breastfeeding was common among mothers interviewed, with high rates of continued breastfeeding at both 1 and 2 years of age.

Several factors have been found to be associated with prelacteal feeding in Nepal. Early initiation of breastfeeding has been found to have a protective effect against prelacteal feeding (Khanal *et al*. 2013a), while additional studies have indicated that delivery by caesarean section can delay the initiation of breastfeeding and therefore may be associated with higher rates of prelacteal feeding (Khanal & Sauer 2013; Pandey *et al.* 2010). In this study, vaginal deliveries, as compared with C‐section deliveries, were associated with higher rates of early initiation of breastfeeding (49.3% vs. 20.2% for discharged mothers, *P* < 0.001), and mothers who delivered their newborn by C‐section reported higher rates of prelacteal feeding, as compared with mothers who delivered vaginally (89.9% vs. 42.8%, *P* < 0.001). Among mothers interviewed, rates of C‐section delivery were high, reaching almost 30%, which may be one contributing factor to the high rates of prelacteal feeding. Following this survey, additional qualitative interviews were conducted among clinical staff in nine Kathmandu Valley hospitals, during which many doctors and nurses reported difficulties with initiating breastfeeding soon after caesarean deliveries, including separation of mother and infant after surgery because such mothers recuperate in general wards and infants are not permitted there because risk of infection (I. Adhikary, unpublished observations). Research has also found that early *post‐partum* skin‐to‐skin contact between mothers and newborns has a powerful influence over the duration of exclusive breastfeeding (Vaidya *et al*. 2005) and early initiation of breastfeeding among mothers delivering by caesarean section (Stevens *et al*. 2014); only 9% of discharged mothers reported immediately holding their newborn after delivery.

Various studies have also highlighted the important role that health workers' advice and counselling plays in influencing infant and young child feeding practices employed by mothers in Nepal. One study found that information on optimal feeding practices provided by health workers to mothers during child immunization visits was significantly associated with appropriate feeding practices by these mothers (Chapagain 2013). Several studies have illustrated the association between ANC visits and IYCF practices, whereby exclusive breastfeeding and complementary feeding practices were found to improve with the number of ANC visits received by mothers (Karas *et al*. 2012; Joshi *et al*. 2012; Khanal & Sauer 2013; Khanal *et al*. 2013a). A study conducted within Tribhuvan University Teaching Hospital in Kathmandu Valley assessed newborn care knowledge and practices of 100 *post‐partum* mothers; health personnel, especially nurses, were found to have played a large role in encouraging mothers to initiate breastfeeding within 1 hour (Shrestha *et al*. 2013).

The influential role of health workers' can also result in suboptimal infant and young child feeding practices if the messages contradict recommendations for optimal practices; specifically, positive breastfeeding counselling and advice can be negated by health workers' encouragement of prelacteal feeding. In this survey, a strong association was found between health workers' recommendations of breastmilk substitutes and mothers prelacteal feeding; mothers that had received a recommendation to use breastmilk substitute from a health worker were 16.7 times more likely to provide a prelacteal feed to their newborn as compared with mothers that did not receive a recommendation. Prior studies have also noted the influential role of prelacteal feeding recommendations by health workers in increasing rates of prelacteal feeding (Isenalumhe & Oviawe 1987; Hossain *et al*. 1992; Talukder *et al*. 1997); one study found that urban Nepali mothers whose births were attended by a health professional had a greater risk of bottle feeding their infants (Pandey *et al*. 2010).

Because interviews were conducted with mothers and not health workers, the reason for such high rates of breastmilk substitutes recommendations by health workers, particularly for prelacteal feeding, is not entirely clear. One study from Nigeria assessed reasons why health workers encouraged mothers to provide prelacteal feeds to their newborns; nurses were found to be most likely to recommend prelacteal feeding because of theorized low milk production in the first days after delivery (Akuse & Obinaya 2002). In this survey in Kathmandu Valley, nurses accounted for the majority of prelacteal feeding recommendations to mothers at discharge after delivery. Almost all mothers who prelacteal fed reported doing so because their milk had not yet come in or because they believed their milk supply was low; the impact of this concern of insufficient milk supply on cessation of exclusive breastfeeding has been noted in other research in Nepal (Ulak *et al*. 2012). The qualitative interviews conducted after this survey in Kathmandu Valley hospitals also found that many nurses and doctors reported mothers' milk supply to be inadequate in the first day after delivery and the need to provide infant formula during this time (I. Adhikary, unpublished observations). It is plausible that both mothers and health workers shared a common concern regarding low milk supply soon after delivery, and perhaps also a lack of recognition that colostrum, while produced in small amounts, is sufficient in the first days after delivery, which led to high rates of breastmilk substitute recommendations by health workers and subsequent prelacteal feeding by mothers. While this is a common concern among mothers, the milk supply of most mothers is adequate for newborns, particularly if breastfeeding is initiated soon after delivery. Given the importance of early initiation of breastfeeding in stimulating milk production, the encouragement of prelacteal feeding by health workers for fear of low milk supply could actually result in decreasing a mother's supply that would otherwise be sufficient (Yamauchi and Yamanouchi 1990; Ahmed *et al*. 1996; WHO 1996).

Findings from this survey also indicate that mothers' exposure to breastfeeding advice before delivery may be low in Kathmandu Valley; only 11.6% (*n* = 35) of mothers discharged after delivery reported receiving such advice during their ANC visits. Increasing mothers' exposure to breastfeeding advice during antenatal care could ensure that messages regarding exclusive breastfeeding are heard before a child is born. Additionally, addressing mothers' fears about insufficient milk supply and health workers' misconceptions about milk production in the first few days after delivery, as well as emphasizing that colostrum is all a newborn needs in the first days of life, may help to reduce the early introduction of other foods or liquids.

Lack of breastfeeding support skills and knowledge among health workers may result in mothers thinking their breastfeeding problems are because of a lack of milk and lead to health workers recommending infant formula. The provision of lactation management training, particularly around encouragement of early initiation of breastfeeding, early skin‐to‐skin contact, the importance of colostrum and introduction of complementary foods at 6 months, may bolster health workers' practical knowledge and skills and could reduce the high rates of prelacteal feeding and increase the duration of exclusive breastfeeding among mothers living in Kathmandu Valley. Additionally, given the high rate of caesarean section deliveries found in these Kathmandu Valley hospitals, specific breastfeeding counselling for mothers who have delivered by caesarean section would be beneficial. The reinforcement and expansion of the Baby Friendly Hospital Initiative may also improve breastfeeding counselling by increasing the number of trained hospital staff in Kathmandu Valley. Four hospitals in Kathmandu Valley were designated as BFHI in 1997–1998; however, a 2012 assessment showed that none were still fully compliant with the all BFHI criteria (Shrestha *et al*. 2012).

The main limitations of this study relate to the nature of conducting a facility‐based survey. The main objective of the study was to assess promotions within Kathmandu Valley's health system; therefore, interviews were conducted within health facilities in order to ensure access to a study population that was currently utilizing healthcare within this system. However, for assessment of early breastfeeding practices and utilization of breastmilk substitutes among the general population of Kathmandu Valley, this presented a limitation. These practices remain unknown among mothers who deliver at home; in 2011, 27.9% of women in urban Nepal reported delivering their last child at home (MOHP 2012). Finally, the cross‐sectional design of this research limits the ability to establish causality between exposure to promotion and utilization of breastmilk substitutes among mothers.

## Source of funding

This research was funded by the Bill & Melinda Gates Foundation.

## Conflicts of interest

The authors have no conflicts of interest to declare.

## Contributions

AP analyzed the data and prepared the manuscript. SH and EZ conceptualized and designed the study, with input from SU. MC oversaw questionnaire development and technology for data collection. IA and SD managed data collection. All authors reviewed and provided input on the final article.
